# The Presence of Ovarian Cancer and the Incidence of Subsequent Open-Angle Glaucoma: A Population-Based Cohort Study

**DOI:** 10.3390/cancers16162828

**Published:** 2024-08-12

**Authors:** Chia-Yi Lee, Shun-Fa Yang, Yu-Ling Chang, Jing-Yang Huang, Chao-Kai Chang

**Affiliations:** 1 Institute of Medicine, Chung Shan Medical University, Taichung 402, Taiwan; 2 Nobel Eye Institute, Taipei 100, Taiwan; 3 Department of Ophthalmology, Jen-Ai Hospital Dali Branch, Taichung 412, Taiwan; 4 Department of Medical Research, Chung Shan Medical University Hospital, Taichung 402, Taiwan; 5 Department of Medical Education, Cathay General Hospital, Taipei 106, Taiwan; 6 Department of Optometry, Da-Yeh University, Chunghua 515, Taiwan

**Keywords:** ovarian cancer, open-angle glaucoma, oxidative stress, age, epidemiology

## Abstract

**Simple Summary:**

Ovarian cancer is a prevalent gynecological cancer and a leading cause of cancer-associated mortality worldwide. Ovarian cancer is related to several oxidative stress-related diseases. Open-angle glaucoma (OAG) is a neurodegenerative disease that is associated with elevated oxidative stress, while the relationship between OAG and ovarian cancer is vague. Consequently, we aim to survey the interrelationship between ovarian cancer and OAG. In this retrospective cohort study, people with ovarian cancer were recruited and age-matched with a 1:4 ratio of non-ovarian to cancer. The multivariable analysis exhibited a higher incidence of OAG in the ovarian cancer group than the non-ovarian cancer patients, which was more prominent in those older than 60 years and with ovarian cancer for longer than two years. Thus, the presence of ovarian cancer is associated with a higher possibility of later OAG. Routine glaucoma exams could be recommended for women diagnosed with ovarian cancer for longer than 2 years or who are older than 60.

**Abstract:**

We aim to explore the possible association between ovarian cancer and the subsequent development of open-angle glaucoma (OAG) using the Taiwan Longitudinal Health Insurance Database (LHID) 2000. A retrospective cohort study was executed, and individuals with ovarian cancer were enrolled and age-matched (with a 1:4 ratio) to non-ovarian cancer individuals. A total of 4990 and 19,960 patients were put into the ovarian cancer and control groups. The main outcome was the presence of OAG according to the LHID 2000 codes. The Cox proportional hazard regression was adopted to demonstrate the adjusted hazard ratio (aHR) and 95% confidence interval (CI) of OAG between the ovarian cancer and control groups. There were a total of 241 and 1029 OAG cases observed in the ovarian cancer group and the control group, respectively. The incidence of OAG was significantly higher in the ovarian cancer group than in the control group according to multivariable analysis (aHR: 1.18, 95% CI: 1.02–1.37, *p* = 0.022). The ovarian cancer patients older than 60 years showed a significantly higher risk of OAG compared to the non-ovarian cancer individuals of the same age (aHR: 1.39, 95% CI: 1.16–1.63, *p* = 0.001). Additionally, ovarian cancer individuals with a disease interval of more than two years presented a significantly higher incidence of OAG than the non-ovarian cancer group (*p* < 0.05). In conclusion, ovarian cancer positively correlates with a high rate of subsequent OAG, especially in elderly persons with a long disease interval.

## 1. Introduction

Ovarian cancer is a prevalent gynecological cancer that is the fifth leading cause of cancer-associated mortality among the female population of America [1,2]. The usage of hormone replacement therapy, late childbearing, oral contraceptive employment, ovarian cancer family history, early menarche, obesity, and preceding endometriosis are known risk factors for ovarian cancer formation [1,3,4]. The current approaches for ovarian cancer include both surgery and traditional chemotherapy, while bevacizumab has gained attention in the past 10 to 20 years [5,6]. Although multiple treatments can be applied, the ovarian cancer prognosis is guarded, and the five-year survival rate cannot reach 50 percent [7].

The existence of ovarian cancer correlates to certain disorders [8,9]. In a previous study, ovarian cancer occurred in individuals with a greater body mass index or obesity more regularly [4]. Also, type 2 diabetes mellitus (T2DM) is robustly associated with a significantly bad prognosis of ovarian cancer [10]. Furthermore, certain metabolic syndromes, like hyperlipidemia, present a growing risk of ovarian cancer instance [11]. On the other hand, dementia is an illness of oxidative stress, which develops in more than 2 percent of ovarian cancer patients [12,13].

Glaucoma is a neurodegenerative ocular disease that mainly involves retinal ganglion cell death and subsequent glaucomatous optic neuropathy [14]. Elevated intraocular pressure is the crucial risk factor for open-angle glaucoma (OAG) occurrence, and several medications were invented to manage OAG [15,16]. Also, OAG relates to certain systemic diseases like cardiovascular disorders and T2DM [17,18], while the relationship between ovarian cancer and OAG remains vague. Since both ovarian cancer and glaucoma are associated with high oxidative stress [19,20], a positive relationship between them might be present.

As a result, the objective of this study is to survey the potential correlation between the existence of ovarian cancer and the rate of subsequent OAG using data derived from the Taiwan National Health Insurance Research Database (NHIRD). The subgroup analyses based on age and the disease interval of ovarian cancer were also conducted.

## 2. Materials and Methods

### 2.1. Data Resource

The Taiwan NHIRD restores the medical information of nearly 23 million persons who resided in Taiwan from 1 January 2000 to 31 December 2020. Available medical records in the NHIRD involve the International Classification of Diseases Ninth Revision (ICD-9) diagnostic code, the International Classification of Diseases Tenth Revision (ICD-10) diagnostic code, age, sex, socioeconomic condition, medical exam codes, the physician department codes, procedure/surgery codes, and the international ATC codes for drugs and prescriptions. It is worth noting that medical exams, procedures, and medication provided by the Taiwan National Health Insurance service can be taken from the Taiwan NHIRD. The Taiwan Longitudinal Health Insurance Database (LHID) 2000 was utilized for the analysis in this study, and it is one of the sub-databases of the Taiwan NHIRD. The LHID 2000 holds nearly two million persons who were randomly taken out from the Taiwan NHIRD by the automated software program, and the information in the LHID 2000 is exactly the same as the information in the Taiwan NHIRD.

### 2.2. Subject Selection

**Figure 1:**
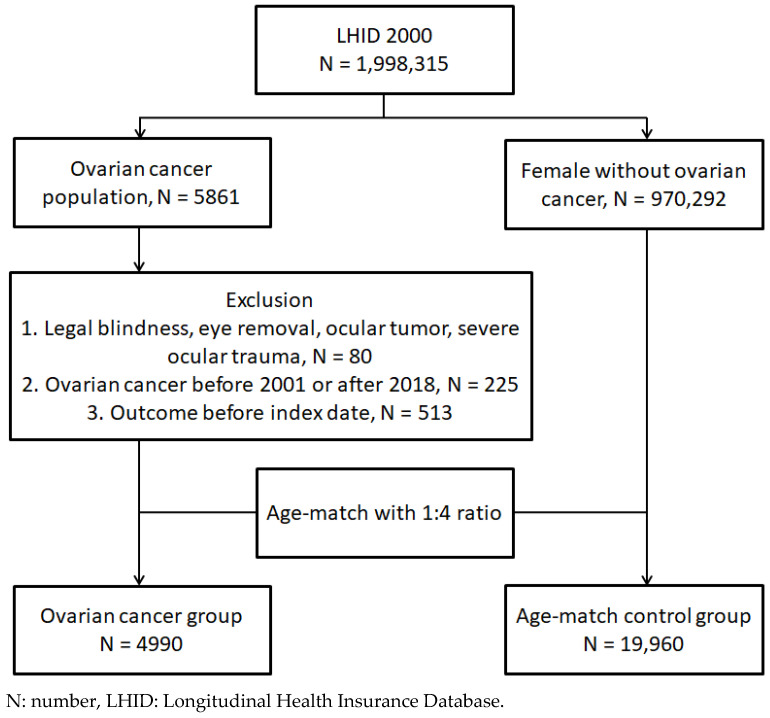
The flowchart of subject selection.

A retrospective cohort study was implemented. Individuals in our LHID 2000 were defined as having ovarian cancer if they filled the succeeding situations: (1) the arrangement of a pelvic exam before the day of the ovarian cancer diagnosis via the procedure codes, (2) the arrangement of computed tomography, a pelvic ultrasound exam, or a cancer antigen 125 test before the ovarian cancer diagnosis by procedure codes, (3) ovarian cancer diagnosis via the corresponding ICD-9 and ICD-10 diagnostic codes, and (4) the ovarian cancer was diagnosed in a gynecological clinic. The index date was designated as six months after the emergence of the ovarian cancer. In addition, these exclusion criteria were implemented: (1) the ocular tumor was present before the index date via the related ICD-9 and ICD-10 diagnostic codes, (2) legal blindness was present before the index date via ICD-9/ICD-10 diagnostic codes, (3) severe ocular trauma occurred before the index date via the related ICD-9 and ICD-10 diagnostic codes, (4) the eye removal status was found before the index date via the related procedure codes, (5) the primary outcome (i.e., OAG) was found before the index date, and (6) ovarian cancer was diagnosed before 2001 or after 2018 for standardizing the timeliness of the ovarian cancer exposure. After that, one ovarian cancer person was age-matched to four non-ovarian cancer participants, and the latter group was regarded as the control group. Finally, a total of 4990 and 19,960 patients were gathered in the ovarian cancer and control groups, respectively. The flowchart of subject selection is represented in Figure 1.

### 2.3. Main Outcome

The main outcome of this study is the occurrence of OAG via the following factors: (1) the diagnosis of glaucoma via the related ICD-9 and ICD-10 codes, (2) optical coherence tomography, direct fundoscopy, or a visual field exam was completed before the OAG diagnosis by procedure codes, (3) the administration of anti-glaucomatous medications after OAG emergence via the related ATC/medication codes, and (4) the OAG diagnosis was established by an ophthalmologist. To better assemble the time sequence between ovarian cancer and the ensuing OAG development, only the OAG cases found after the index date were deemed as achieving the outcome. Regarding the follow-up period of the main outcome, the follow-up for patients in this study would be ended until the occurrence of OAG, the patient left the national health insurance system, or the end date of the NHIRD and LHID 2000 (31 December 2020).

### 2.4. Potential Covariates

To better evaluate the outcome between groups, several covariates were put in the statistical analysis to adjust for the possible influence of these covariates on OAG occurrence: age, occupation, hypertension, coronary heart diseases, T2DM, dyslipidemia, ischemic stroke, hemorrhage stroke, peripheral vascular disease, corticosteroid usage, uveitis, and diabetic retinopathy. The presence of the above covariates was judged via the related demographic codes, the ICD-9 and ICD-10 diagnostic codes, the procedure codes, and the ATC codes in the LHID 2000. Moreover, only the disorders or corticosteroid usage that was recorded for more than two years in the LHID 2000 were included as covariates to ensure that the intervals of the covariates are adequate to affect the OAG occurrence.

### 2.5. Statistical Analysis

The SAS version 9.4 (SAS Institute Inc., Cary, NC, USA) was assigned for the statistical analyses included in this study. The descriptive analyses were assigned to demonstrate the demographic material, systemic diseases, and drugs of the two groups. The standard mean difference (SMD) or standardized proportion differences was assigned to check the distribution of different covariates between the two groups, and statistical significance was deemed as SMD > 0.1. After that, the Cox proportional hazard regression was assigned to display the adjusted hazard ratio (aHR) and relative 95% confidence interval (CI) of OAG cases between the two groups. The Cox regression is utilized to build a predictive model for the time-to-event data, incorporating the given predictor variables. In this study, we focus on the time to achieve OAG for cancer patients compared with control patients, with the presence of cancer being included as a binary predictor variable. This model aims to elucidate the impact of cancer on the time required to achieve OAG. The influence of age, affair, systemic diseases, and medications were all regulated in the Cox proportional hazard model. Regarding the subgroup analyses of this study, the ovarian cancer subjects were sorted by age (<40 years, 40–60 years, and >60 years) and ovarian cancer interval (<2 years, 2–5 years, and >5 years). Later, the Cox proportional hazard regression was assigned again with modifications for co-morbidities and prescriptions, and the aHR and relative 95% CI were appraised. Statistical significance was specified as *p* < 0.05 in this study, and a *p* value lesser than 0.001 was illuminated as *p* < 0.001.

## 3. Results

The basic features between the two groups are demonstrated in Table 1. The age allocations between the two groups were similar due to the PSM management (SMD: 0.002). Additionally, the type of affair revealed a non-significant difference between these groups (SMD: 0.012). Regarding the co-morbidities and prescriptions, the allocations of T2DM (SMD: 0.387) and coronary heart disease (SMD: 0.125) were both significantly higher in the ovarian cancer group compared to the control group. Nevertheless, the rest of the systemic disorders and the corticosteroid treatment showed similar allocations between the two groups, respectively (Table 1).

There were 241 and 1029 OAG episodes found in the ovarian cancer group and the control group, respectively (Table 2). After the regulation of demographic material, co-morbidities, and prescriptions, the incidence of OAG cases was significantly higher in the ovarian cancer group than in the control group, according to the results of Cox proportional hazard regression (aHR: 1.18, 95% CI: 1.02–1.37, *p* = 0.022) (Table 2).

Concerning the subgroup analysis of age and disease course, the ovarian cancer patients older than 60 years had a significantly higher chance of OAG occurrence compared to the non-ovarian cancer individuals with the same age interval (aHR: 1.39, 95% CI: 1.16–1.63, *p* = 0.001), while the younger ovarian cancer women did not have a higher rate of developing an OAG condition than the non-ovarian cancer population (Table 3). In another subgroup analysis stratified by the ovarian cancer course, the ovarian cancer individuals with a disease course longer than two years represented a significantly higher incidence of an OAG condition than the non-ovarian cancer group (both *p* < 0.05). On the contrary, ovarian cancer women with a disease course of less than two years had a risk of an OAG condition similar to the control group (*p* = 0.379) (Table 3).

## 4. Discussion

In this study, the ovarian cancer group had a significantly higher incidence of OAG than the non-ovarian cancer group. Further, this specific correlation is more significant in ovarian cancer women older than 60 years. Also, women with an ovarian cancer course of more than two years appear to have a higher chance of developing OAG, while the incidence of OAG in those with an ovarian cancer interval of less than two years was not elevated significantly.

Several mechanisms were proposed regarding the formation of ovarian cancer in the literature [21,22,23]. Progesterone and estrogen, which accompany steroid hormone effects, relate to an ovarian environment alteration and ovarian cancer occurrence [24]. Additionally, gene variations containing the BRCA1 gene and BRCA2 gene augment the possibility of a high-degree serous ovarian carcinoma circumstance [25]. Oxidative stress is another leading pathophysiology with respect to the circumstance of ovarian cancer [26,27]. Inducible nitric oxide synthase as well as superoxide dismutase are raised in women with ovarian cancer [20]. Another reactive oxygen species, the antioxidant enzyme NAD(P)H:quinone oxidoreductase 1, could regulate the effectiveness of chemotherapy for handling ovarian cancer [28]. Regarding the genetic ingredient, the antioxidant-related gene NRF2 can predict the prognosis of ovarian cancer, as was found in a past study [29]. On the other hand, glaucoma, including OAG, involves the degeneration of the optic nerve and retinal neurons, which is similar to other neurodegenerative disorders, such as Alzheimer’s disease [14,30]. Moreover, the retinal vascular defect in OAG and NTG was frequently observed [16,31]. In addition to neuron degeneration and vascular damage, the high oxidative stress can contribute to the occurrence of glaucoma, including OAG, and the elevated level of reactive oxygen species can lead to the upregulation of HIF-1a, death of retinal ganglion cells, and subsequent glaucoma [32]. Another previous study also revealed that the application of antioxidant agents, such as valproic acid, can prevent glaucoma deterioration in an animal model [33]. In daily life, dietary antioxidant application may have a preventive effect on the occurrence of glaucoma [34]. Both ovarian cancer and glaucoma share the characteristics of high oxidative stress [19,20,35,36]; thus, the high baseline oxidative stress situation in ovarian cancer may cause a higher incidence of subsequent OAG. The above hypothesis was supported by the findings of this study.

In this study, individuals with ovarian cancer had a significantly higher incidence of a subsequent OAG episode. Previous studies have discussed the correlation between OAG and neoplasm, and the presence of nasopharyngeal carcinoma is associated with a higher risk of OAG development, and the application of androgen deprivation therapy could slightly reduce the incidence of OAG [37,38]. However, the association between OAG and gynecological cancer has not been fully elucidated. To our knowledge, our discovery may provide preliminary evidence that demonstrates the positive correlation between the ovarian cancer condition and the subsequent OAG condition. Moreover, we excluded the OAG condition that occurred before our index date (i.e., 6 months after the diagnosis of ovarian cancer); thus, the time sequence between earlier ovarian cancer and late OAG development could be proposed. In addition, we adjusted several established risk factors for the OAG condition, including age, hypertension, T2DM, some vascular disorders, and corticosteroid treatment, in our Cox proportional hazard regression [16,39]. As a consequence, the attendance of ovarian cancer may be an independent prospect factor for the development of OAG. The high level of oxidative stress can trigger neoangiogenesis and the dissemination of ovarian cancer, which can alter the tumor stage [40], and the antioxidant distributions would be changed in advanced ovarian cancer [41]. Similarly, the high oxidative stress would speed up the progression of glaucoma [42], and the serum antioxidant expression would be decreased in a specific type of glaucoma [43]. Together with other oxidative stress-related mechanisms that we discussed in the earlier sections [19,20,35,36], ovarian cancer and the associated high oxidative stress could relate to consecutive glaucoma in multiple respects, and the results of our clinical research supported the findings from previous experimental studies with adequate rationality. Although the complete percentage of OAG cases was numerically lower in the ovarian cancer group than in the control group, the complete follow-up person-month was likewise longer in the control group. The only imaginable explanation is that ovarian cancer is a disease with high mortality and a lower five-year survival rate [7], so the average lifespan in the ovarian cancer group was shorter than that in the control group. If the average follow-up period of ovarian cancer was longer, the overall OAG percentage could be higher than the OAG percentage in the control group. However, a further study with an adequate follow-up interval is needed to verify this conception.

In the age-based subgroup analysis, the ovarian patients older than 60 years demonstrated a significantly higher incidence of OAG development compared to the control group. Age was a risk factor for the development of OAG in a previous study in which OAG tended to occur in patients older than 60 years [16]. Also, an increasing OAG risk of 2.0- to 2.5-fold per 10 years of age was found after the age of 40 years [39]. On the other hand, ovarian cancer commonly occurs after 60 years old, with an incidence of more than 30 cases per 100,000 women [3]. Besides the effects of these two diseases, old age itself is associated with elevated oxidative stress, which may result from the dysfunction of mitochondria with respect to adapting to oxidative stress [44]. Consequently, it is possible that the elderly ovarian cancer patients had higher baseline oxidative stress levels and that OAG occurs more easily in them compared to their younger counterparts, which was proven by the results of this study at least to some degree. Additionally, another subgroup analysis based on the duration of ovarian cancer illustrated a higher incidence of OAG in ovarian cancer patients with a disease interval of 2–5 years and more than 5 years compared to the non-ovarian cancer population. Studies have rarely tried to evaluate this issue. In a previous study, the duration of nasopharyngeal carcinoma was associated with a higher incidence of OAG, which may be due to persistent inflammation [37]. Also, ovarian cancer progresses as time passes, which may be associated with higher oxidative stress. Accordingly, the incidence of OAG may be higher under the prolonged expression of high oxidative stress in ovarian cancer patients with a long disease interval. In this study, the possible cut-point of a significant risk of OAG in the ovarian cancer population was two years, which might serve as a reference for ophthalmic referrals.

In the field of epidemiology, ovarian cancer is a very frequent gynecological cancer throughout the world, which is similar to breast cancer [7]. More than 230,000 individuals suffered from ovarian cancer in 2018 [7], and more than 90,000 women die due to ovarian cancer each year [1]. Regarding the high-prevalence areas, North America, Oceania, and Europe presented the highest mortality rates that were caused by ovarian cancer [45]. Glaucoma, including OAG, is also a common ophthalmic disorder, and more than 50 million people have been diagnosed with glaucoma [16], and the number of all glaucoma individuals is estimated to be 111 million by the year 2040 [15]. The influence of glaucoma on public health issues is huge in Asian countries, which is the leading reason for irreversible blindness as well as severe visual impairment [46,47]. Moreover, approximately 7 million patients experience blindness due to glaucoma [14]. Because both ovarian cancer and glaucoma are major disorders and can lead to significant burdens for humans, it would be valuable to understand any relationships between them.

Certain limitations existed in this study. In the first place, both the NHIRD and LHID 2000 are claimed to be datasets that only sustain the codes of diagnosis, management, and prescription without real medical scripts. As a consequence, compelling information, including the severity of systemic diseases, the medical compliance of corticosteroids, the site/size/laterality of ovarian cancer, the results of ultrasound and computed tomography for ovarian cancer, the cancer antigen 125 level for ovarian cancer, the site of lymph node involvement if relevant, the definite treatment dose of chemotherapy if practiced, the treatment efficacy of ovarian cancer, the recurrence of ovarian cancer if relevant, the intraocular pressure of OAG, the consequences of optical coherence tomography and visual field test for OAG if completed, the configuration of the optic disc, the treatment efficacy of OAG, and the deterioration of OAG, cannot be appraised or cannot be fully appraised. In the second place, the retrospective architecture of this study will dwindle the homogeneity of the two groups even though we matched them via age and conducted multivariable analyses to adjust for the effect of each covariate. In addition, we did not separate ovarian cancer into different subtypes because certain types of ovarian cancer, like malignant germ cell ovarian tumors, are too rare in the NHIRD/LHID 2000 to conduct an analysis. Finally, the different corticosteroids have different potencies for intraocular pressure elevation and glaucomatous optic neuropathy, but we cannot separate them because many patients take multiple corticosteroids.

## 5. Conclusions

In conclusion, ovarian cancer patients have a significantly higher incidence of subsequent OAG after adjusting for multiple risk factors for glaucoma. Furthermore, this correlation is more prominent in those older than 60 years and who have been diagnosed with ovarian cancer for more than two years. Consequently, routine glaucoma examinations might be recommended for ovarian cancer individuals with prolonged disease intervals or who are elderly. Further large-scale prospective studies are needed to appraise whether the treatment efficacy of ovarian cancer would disturb the therapeutic result of OAG via the application of computational methods such as deep neural networks.

## Figures and Tables

**Table 1:** Baseline feature in the whole study cohorts.

Features	Control Group (N: 19,960)	Ovarian Cancer Group (N: 4990)	SMD
Age			0.002
<40	5964 (29.88%)	1489 (29.86%)	
40–49	5125 (25.69%)	1266 (25.39%)	
50–59	4097 (20.53%)	1038 (20.78%)	
60–69	2367 (11.84%)	588 (11.81%)	
70–79	1566 (7.83%)	399 (7.96%)	
≥80	841 (4.23%)	210 (4.20%)	
Occupation			0.012
Government employee	1457 (7.31%)	438 (8.79%)	
Worker	12,068 (60.45%)	3044 (60.98%)	
Farmer and fisherman	2967 (14.87%)	643 (12.90%)	
Low-income	94 (0.47%)	28 (0.56%)	
None	2919 (14.63%)	726 (14.54%)	
Others	455 (2.28%)	111 (2.22%)	
Co-morbidity			
Hypertension	3230 (16.18%)	936 (18.75%)	0.042
T2DM	1527 (7.65%)	465 (9.32%)	0.387 *
Coronary heart disease	747 (3.74%)	234 (4.69%)	0.125 *
Dyslipidemia	1687 (8.45%)	446 (8.94%)	0.034
Ischemic stroke	1561 (7.82%)	411 (8.24%)	0.041
Hemorrhage stroke	303 (1.52%)	77 (1.54%)	0.001
Peripheral vascular disease	90 (0.45%)	23 (0.46%)	0.001
Corticosteroids usage	3818 (19.13%)	1020 (20.45%)	0.064
Uveitis	27 (0.14%)	11 (0.22%)	0.018
Diabetic retinopathy	76 (0.38%)	27 (0.54%)	0.020

T2DM: type 2 diabetes mellitus, N: number, SMD: standard mean difference. * denotes a significant difference between groups.

**Table 2:** Risk of open-angle glaucoma between the two groups.

OAG Event	Control Group (N: 19,960)	Ovarian Cancer Group (N: 4990)	*p* Value
Follow-up person-month	2,190,334	442,252	
Case	1029	241	
Crude hazard ratio	Reference	1.16 (1.01–1.34)	
aHR	Reference	1.18 (1.02–1.37) *	0.022 *

aHR: adjusted hazard ratio, CI: confidence interval, N: number, OAG: open-angle glaucoma. * denotes a significant difference after adjusting for demographics and co-morbidities.

**Table 3:** Subgroup analysis stratified by age and ovarian cancer duration.

Subgroup	aHR ^#^	95% CI	*p* Value
Age			
<40 years	0.95	0.76–1.19	0.641
41–60 years	1.07	0.93–1.24	0.403
>60 years	1.39	1.16–1.63	0.001 *
Ovarian cancer duration			
<2 years	1.15	0.96–1.28	0.379
2–5 years	1.23	1.01–1.47	0.038 *
>5 years	1.21	1.03–1.42	0.035 *

aHR: adjusted hazard ratio, adjusted for demographics and co-morbidities, CI: confidence interval. * Denotes a significant difference between groups. ^#^ Risk of open-angle glaucoma in ovarian cancer group compared to non-ovarian cancer group.

## Data Availability

The original data used in this study are not available due to the policy of the Taiwan National Health Insurance Administration.
